# RNA-seq reveals role of cell-cycle regulating genes in the pathogenicity of a field very virulent infectious bursal disease virus

**DOI:** 10.3389/fvets.2024.1334586

**Published:** 2024-02-01

**Authors:** Jinnan Chen, Weiwei Wang, Shangquan Li, Zhiyuan Wang, Wenbo Zuo, Tingbin Nong, Yihai Li, Hongquan Liu, Ping Wei, Xiumiao He

**Affiliations:** ^1^Guangxi Key Laboratory for Polysaccharide Materials and Modifications, School of Marine Sciences and Biotechnology, Guangxi Minzu University, Nanning, China; ^2^Institute for Poultry Science and Health, Guangxi University, Nanning, China

**Keywords:** infectious bursal disease virus, RNA-seq, proteomic, chicken, thymus, pathogenesis

## Abstract

Infectious bursal disease virus (IBDV) infection causes highly contagious and immunosuppressive disease in poultry. The thymus, serving as the primary organ for T cell maturation and differentiation, plays an important role in the pathogenicity of IBDV in the infected chickens. However, there are no reports on the molecular pathogenesis of IBDV in the thymus currently. The aim of the study was to elucidate the molecular mechanisms underlying the pathogenicity of a field very virulent (vv) IBDV strain NN1172 in the thymus of SPF chickens using integrative transcriptomic and proteomic analyses. Our results showed that a total of 4,972 Differentially expressed genes (DEGs) in the thymus of NN1172-infected chickens by transcriptomic analysis, with 2,796 up-regulated and 2,176 down-regulated. Meanwhile, the proteomic analysis identified 726 differentially expressed proteins (DEPs) in the infected thymus, with 289 up-regulated and 437 down-regulated. Overall, a total of 359 genes exhibited differentially expression at both mRNA and protein levels, with 134 consistently up-regulated and 198 genes consistently down-regulated, as confirmed through a comparison of the RNA-seq and the proteomic datasets. The gene ontology (GO) analysis unveiled the involvement of both DEGs and DEPs in diverse categories encompassing cellular components, biological processes, and molecular functions in the pathological changes in IBDV-infected thymus. The Kyoto Encyclopedia of Genes and Genomes (KEGG) pathway analysis revealed that the host mainly displayed severely disruption of cell survival/repair, proliferation and metabolism pathway, meanwhile, the infection triggers antiviral immune activation with a potential emphasis on the MDA5 pathway. Network inference analysis identified seven core hub genes, which include *CDK1, TYMS, MCM5, KIF11, CCNB2, MAD2L1*, and *MCM4*. These genes are all associated with cell-cycle regulating pathway and are likely key mediators in the pathogenesis induced by NN1172 infection in the thymus. This study discovered dominant pathways and genes which enhanced our understanding of the molecular mechanisms underlying IBDV pathogenesis in the thymus.

## 1 Introduction

Infectious bursal disease (IBD) is an acute, highly contagious and immunosuppressive viral disease in poultry, and threatens the poultry industry throughout the world (1). The causative agent, Infectious bursal disease virus (IBDV), belongs to the *Avibirnavirus* of the family *Birnaviridae* and characterized by destruction of lymphoid organs in 3- to 6-week-old chickens. Among IBDV strains, serotype 1 IBDV stands out for its pathogenicity in chickens, various subtypes/strains categorized by increasing virulence levels, ranging from mild (attenuated) to intermediate, classical, antigenic variants (av), and very virulent (vv) (2). Despite extensive use of live and inactivated vaccines to combat IBD, the emergence of vv, av., and reassortment IBDV strains in recent years has added complexity to IBD epidemiology in the field (3–5). This suggests that there is still a persistent challenge in prevention and control of IBD. To date, the molecular basis of pathogenicity, the immunosuppression and the exact cause of clinical disease and death of IBDV remains incomplete. Therefore, a further insight into IBDV pathogenesis is thus essential for effectively managing and preventing this disease.

The principal target for IBDV is Ig-M bearing B cells in the bursa of Fabricius (BF). However, IBDV infection can affect all compartments of a bird’s immune system (6). A serotype 1 virus has been isolated from susceptible chicks with lymphocyte depletion observed in the BF, spleen, and/or thymus. After virus infection, the incubation period is very short (2–3 days). Virus infection leads to a rapid and progressive loss of B cells in the bursal, thymic medulla and peripheral blood (7). While T cells, although are not infected by IBDV, play a crucial role in modulating the pathogenesis of IBDV. During the early phase of the disease, these T cells limit viral replication in the BF, promote bursal tissue damage, and delay tissue recovery (8). Both CD4+ and CD8+ T cells have been confirmed to infiltrate the BF after infection (9). IBDVs differing in virulent also have been showed to cause differential disruption of T-cell system (10). Thus, being the main organ for the maturation and differentiation of avian T lymphocytes, the thymus holds particular significance in the pathogenicity of IBDV. In a previously study (11), we found that IBDV field isolates of different genotypes induced varying degree of pathogenicity in the thymus of the commercial Three-Yellow chickens. More virulent strains exhibited pronounced thymic oedema at 3 days post infection (dpi), followed by atrophy at 7dpi, accompanied by T cell depletion in the thymus. In another study, Inoue et al. (12) reported that different virulent strains of IBDV induced follicular lymphoid necrosis and depletion in the thymus of SPF chickens in a virulence-dependent manner, confirming the virus’s persistence in the thymus. Williams and Davison (10) confirmed that vvIBDV strain UK661 infection led to more severe clinical syndromes, characterized by the depletion of Bu-1+ cells in the BF and thymus, compared to classical virulent strains. Additionally, Sharma’s group (13) revealed that thymic atrophy is associated with the acute phase of the disease and may serve as an indicator of the virulence of IBDV isolate, and the severity of pathology following UK661 infection greatly exceeded the ones of the less virulent strains, with the thymus pathology progressing much further (14). These findings collectively underscore the important role of thymic pathogenicity in contributing to the immunosuppression/pathology caused by IBDV in the infected chickens. While the BF has been confirmed to be one of the most important target organ for IBDV, numerous researches have focused on deciphering the pathogenicity mechanisms of IBDV by examining changes in the BF or *in vitro* cultured immune cells (15–23). However, there are no reports on the molecular pathogenesis of IBDV in the thymus currently. As a high-throughput technologies, Omics techniques, such as RNA-sequencing (RNA-seq) and quantitative proteomics, have been instrumental in deciphering the intricate biological processes underlying complex diseases (24). In contrast to analyzing transcript and protein changes separately in response to environmental insults, the integrated analysis of these datasets proves to be a powerful tool for validating significant expression changes and elucidating whether transcript abundance influences protein alterations. This integrated approach holds substantial potential for offering a more comprehensive understanding of the gene expression regulation involved in specific diseases (25). The purpose of the study was to elucidate the molecular mechanisms underlying the pathogenicity of a field very virulent (vv) IBDV strain NN1172 in the thymus of SPF chickens using integrative transcriptomic and proteomic analyses. The findings would lay a foundation for further studies on a comprehensive understanding of IBDV pothogenicity in chicken.

## 2 Materials and methods

### 2.1 Virus

The virus strain NN1172 (HLJ0504-like vvIBDV) was a field strain and has been identified to be a naturally reassortant IBDV (*v*VP2 gene from vvIBDV and VP1-b from distinct origin which is similar to an earlier Chinese vvIBDV strain HLJ0504) by our group (3, 11). NN1172 was propagated in 9-day-old SPF embryonated chicken eggs and titrated using the methods previously described (26). The titer of virus used for infection was 10^4^ EID_50_ per 0.2 mL per chicken.

### 2.2 Chicken embryos and chickens

SPF chicken eggs were purchased from Beijing Boehringer Ingelheim Vital Biotechnology Co., Ltd. (Beijing, China). 1-day-old SPF chicks were obtained by incubating SPF eggs in an incubator for 21 days. All the SPF chickens were transferred to the isolation facility and no vaccination was administered.

### 2.3 Experiment design

**Figure 1:**
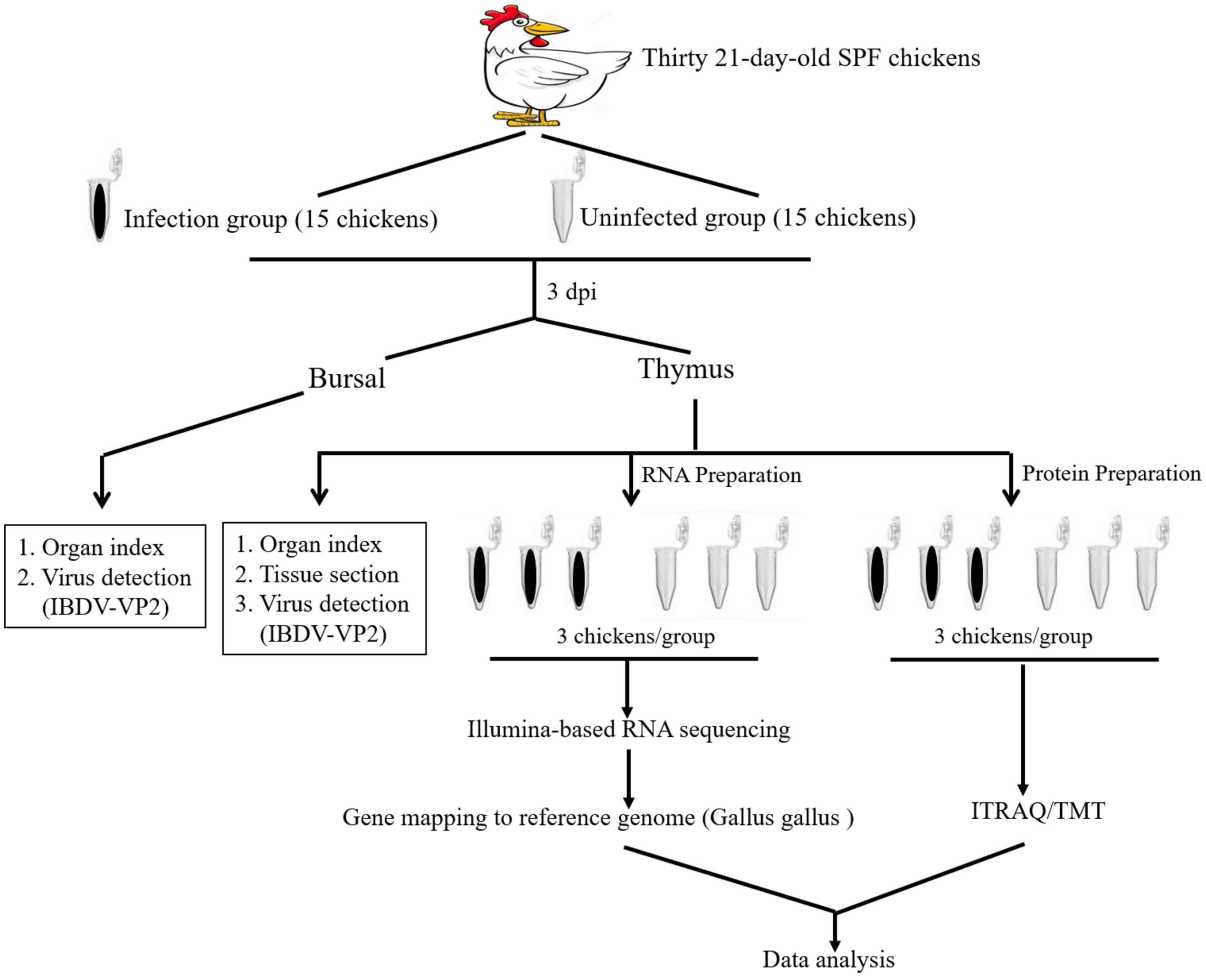
An overview of the transcriptomic and proteomic experiment. Schematic diagram of workflow of the RNA-seq transcriptomic and iTRAQ /TMT technique-based proteomic experiments. Fifteen 21-day-old SPF chickens in infected group received IBDV field strain NN1172. Fifteen chickens served as uninfected group control. The thymus and bursal of each chicken were harvested at 3 days post infection (dpi) and detected as the followings: IBDV nucleotide detection, the immune organ (Bursa or Thymus)/Body Weight index (BBIX) (*n* = 15), histopathological of the thymus, illumina-based RNA sequencing (3 replicates/group) and proteomic based on the iTRAQ/TMT technique of the thymus (3 replicates/group).

Thirty 21-day-old SPF chickens were divided into 2 groups with 15 chickens each as described in Figure 1. Briefly, chickens in infected group received orally a titer of 10^4^ EID_50_/0.2 mL IBDV field strain NN1172. Another 15 chickens were used as the uninfected group and treated with the same dose of 0.2 mL PBS. The thymus and bursal of each chicken were harvested at 3 dpi and detected as the followings: viral determination by vVP2-based RT-PCR, the immune organ (Bursa or Thymus)/Body Weight index (BBIX) (*n* = 15), histopathological of the thymus, illumina-based RNA sequencing and proteomic based on the iTRAQ/TMA technique. Finally, RT-qPCR/western-blotting was used to confirm the differential expression of the genes/proteins.

### 2.4 IBDV genome detection by RT-PCR amplification

The BF/thymus from birds of infected and uninfected group were subjected to RNA isolation by using TRIzol (CWBIO, Beijing, China) according to the manufacturer’s instructions. Briefly, the tissue samples were homogenized in sterile physiological saline solution (1:5, w/v), the mixture was then frozen and thawed three times and finally centrifuged at 3,000 × g for 10 min, 200 μL of the supernatants were suspended in 1 mL of TRIzol reagent for total RNA isolation. Then 1 μg of the RNA sample was used for cDNA synthesis using the HiFiScript 1st Strand cDNA Synthesis Kit (CWBIO, Beijing, China) according to the manufacturer’s instructions. The RT-PCR detection of virus genome based on IBDV-*v*VP2 was described in our previous study (27) and the primers were listed in Table 1.

**Table 1:** List of primers used in RT-PCR or RT-qPCR.

Genes	Sequence	Product (bp)	Accession no. in GenBank	References
IBDV	F:TCCTCTTGCCACTCTTTC	474	NC_004178.1	Wei et al. (27)
	R:CCCAGAGTCTACACCATA			
TLR3	F:GCAACACTTCATTGAATAGCCTTGAT	92	NM001011691.2	Wang et al. (28)
	R:GCCAAACAGATTTCCAATTGCATGT			
IRF7	F:ACCACATGCAGACAGACTGACACT	146	NM_205372	Bolger et al. (29)
	R:GGAGTGGATGCAAATGCTGCTCTT			
CCL19	F:GCAATGATGAAGGAGCAGTACTCACAC	104	NM_001302168.1	Kim et al. (30)
	R:GAAGAAAGGAAAGAAGGAAGCAACTAAT			
CDK1	F:TCTTCTGCCATTCAAGACGAGTTCTG	184	NM_205314.2	Pertea et al. (31)
	R:GATCCTAGCAGTACCTCTGGAGACC			
CCNA2	F:GCCTTGCCTCATGGACCTTCAC	120	NM_205244.3	Pertea et al. (31)
	R:TCTGGTGCGTCAATAAGCGATACTG			
CMPK2	F:GGTGCTGGACATCCTGGAGAAGT	153	XM_015284945.4	Li and Dewey (32)
	R:GCTGGCGGAGACCTTAACAGAAC			
Mx1	F:TTCACGTCAATGTCCCAGCTTTGC	85	XM_040706300.2	Bolger et al. (29)
	R:ATTGCTCAGGCGTTTACTTGCTCC			
CCNB2	F:GGAGAATGCCGTGACTGGACATAA	153	NM_001004369.2	Conesa et al. (33)
	R:CCTTTCGTAGCC TTTACTGGTGGTT			
PCNA	F:TCTGAGGGCTTCGACACCTA	174	NM_204170.3	Pertea et al. (31)
	R:AACCTTTTCCTGATTTGGTGCTT			
APOA1	F:GAGGTGAAGGAGAAGATCCGGC	127	NM_205525.5	Kim et al. (30)
	R:CCTTCTGCTTGGTGAGCTCCT			
β-actin	F:CAACACAGTGCTGTCTGGTGGTA	205	NM_205518.2	Smoot et al. (34)
	R:ATCGTACTCCTGCTTGCTGATCC			

### 2.5 Histology

Thymus samples were collected and fixed in 4% neutral buffered formalin solution, routinely processed and embedded in paraffin and used for the histopathological observation as described by our group previously (35).

### 2.6 Transcriptome analysis

#### 2.6.1 Total RNA isolation, labeling, and sequencing

Total RNA from thymus samples (three independent samples of each group) were isolated by using TRIzol Reagent (Ambion, Beijin, China) following the manufacturer’s instructions. The concentration and purity of the RNA were assessed by Nanodrop 2000 (Thermo Fisher Scientific, Massachusetts, United States), and RNA integrity was determined by 1% agarose gel electrophoresis. The following procedure such as RNA quality inspection, library construction, and sequencing were assigned to Shanghai Majorbio Bio-pharm Technology Co., Ltd. (Shanghai, China).

#### 2.6.2 Data processing and function annotation

Transcriptome sequencing using the high-throughput sequencing platform Illumina Novaseq 6000, Raw sequencing data were processed using CASAVA software from Illumina to generate files in FASTQ (36). Clean data were obtained by using fastp software^1^ to remove sequencing splice sequences, low quality reads, sequences with high indeterminate base information rate and sequences shorter than 50 nt with Q < 30 at 3’end. Clean reads were then mapped to the reference *Gallus gallus* genome (GCF_000002315.6) using TopHat2 software^2^ (37) and further processed by using StringTie^3^ (38). The clean data were then probed on the transcripts in NCBI protein nonredundant,^4^ Swiss-Prot,^5^ Pfam,^6^ EggNog,^7^ Go,^8^ and Kegg databases^9^ using BLASTX to identify the proteins that had the highest sequence similarity with the given transcripts to retrieve their function annotations.

#### 2.6.3 Differentially expressed genes analysis

RSEM software^10^ (39) was applied for quantifying the frequency of the gene and the Transcripts Per Million (TPM) reads were performed to normalize the expression level of each transcript. Differentially expressed genes (DEG) were calculated based on gene read count data by the DESeq2,^11^ and the screening criteria for DEGs were fold change≥1.5 (up-regulated) or fold change≤1.5 (down-regulated) and *p* value < 0.05.

#### 2.6.4 Go functional annotation and KEGG enrichment

BLAST2GO (40)^12^ was used to get Gene Ontology (GO) annotations of unique assembled transcripts for describing biological processes, molecular functions and cellular components. According to the Kyoto Encyclopedia of Genes and Genomes (KEGG) pathway database, pathway analysis was performed using R software (R version 3.2.3). The enrichment statistics of pathways were analyzed using Fisher’s exact test, *p* ≤ 0.05 considered that this pathway was significantly enriched.

### 2.7 Proteome analysis

#### 2.7.1 Protein extraction and digestion

Thymus from the NN1172-infected and uninfected chickens were collected, respectively, for proteome analysis. Thymus sample was transferred to a shaking tube, then added protein lysis buffer (8 M Urea+1% SDS + protease inhibitor), shaking three times (40 s each) with a Wanbo tissue grinder, then incubated on ice for 30 min (vortex and mix for 5–10 s every 5 min), centrifuge at 16,000 × g for 30 min at 4°C, the supernatant was collected, the concentration of the protein was evaluated by Pierce^™^ BCA Protein Assay Kit (ThermoFisher Scientific, Massachusetts, United States), and the protein quality was assessed by SDS-PAGE electrophoresis. The following procedure such as TMT labeling, High-pH RPLC Fractionation and LC–MS/MS Analysis were assigned to Shanghai Majorbio Bio-pharm Technology Co., Ltd. (Shanghai, China).

#### 2.7.2 Protein identification and quantification

The original Raw file from the mass spectrometer were submitted to the Proteome Discoverer 2.2 software, and were then probed on the NCBI and UniProt databases for data search. The highest score for a given peptide mass was used to identify parent proteins. The parameters for searching proteins were as follows: tryptic digestion with two missed cleavages, carbamidomethylation of cysteines as fixed modification, and oxidation of methionines and protein N-terminal acetylation as dynamic modifications. Peptide spectral matches were validated based on *p*-value at a 1% false discovery rate (FDR).

#### 2.7.3 Differentially expressed proteins analysis

The differentially expressed proteins (DEPs) in the NN1172-infected were identified as compared to uninfected control, the fold change of each protein was calculated based on log_2_ value of the relative abundance between infection and uninfection group. Statistics analysis was conducted using Fisher’s exact test. The *p*-value < 0.05 and fold change (FC) >1.2 or <0.83 were used as the threshold to define the significance of protein expression difference. Then DEPs were further subject to functional analysis according to the GO and KEGG pathway databases, and were enriched into different functions with *p* ≤ 0.05 as a threshold to determine the significant enrichment of DEPs.

#### 2.7.4 Protein–protein interactions network analysis and hub gene selection

The differentially expressed genes in both mRNA level and protein level were selected, then the PPI network of Co-expressed DEGs with a highly reliable filtering condition (score > 0.7) were constructed by an online STRING database. Cytoscape (version 3.9.1) was used to visualize the PPI network (41). In Cytoscape, the top 10 genes were obtained by using three different calculation methods of Degree, MNC, and Closeness in the cytoHubba plug-in (42). After taking the intersection, the Hub genes were determined.

#### 2.7.5 RT-qPCR validation of differentially expressed genes

Based on the differentially expressed genes, 10 genes were selected for RT-qPCR validation to verify the accuracy and confidence of NN1172 infection affecting the differential gene expression level in chicken thymus. RT-qPCR was performed on the single strand cDNA prepared for the RNA-seq, by using SYBR Green Master Mix (CWBIO, Beijing, China) in a Step One Plus (ABI, USA). The protocol for detecting of these genes had been previously established and the primers were listed in Table 1. Briefly, the reaction mixture (20 μL), containing 10 μL SYBR Green Master Mix, 10 μM forward primer, 10 μM reverse primer, 2 μL cDNA, was subjected to the following thermal cycling procedure: 95°C for 30 2 min, 40 cycles of 94°C for 30 s and 60°C for 30 s. After amplification, the specificity results of the RT-qPCR were examined by the melting curve analysis. The relative expression of target gene was normalized to β-actin mRNA using 2^-ΔCt^ method, where ΔCt = Ct_target gene_ – Ct_β-actin gene_.

### 2.8 Western blot analysis

Approximately 50 μg total proteins per sample was separated on 12% SDS polyacrylamidegels and then were transferred onto 0.45 μM PVDF membranes (Solarbio, Beijin, China). The proteins on the membrane were blocked with 5% skim milk for 1.5 h at room temperature and then incubated overnight at 4°C with primary antibodies STAT1 (Absin, Shanghai, China), IRF7 (Bioss, Beijing, China), CDK1 (Abclonal, Wuhan, China) and β-actin (Solarbio, Beijin, China). After washing with TBST three times, the membranes were incubated with goat anti-rabbit IgG or goat anti-mouse IgG secondary antibodies conjugated with horseradish peroxidase for 1.5 h, incubated with ECL (electrochemiluminescence) Western Blotting Substrate Kits (Biosharp, Anhui, China). Finally, using β-actin as a reference protein, the bands were analyzed using the Image J software.

### 2.9 Statistical analysis

There were three biological replicates in all experiments. Data were expressed as means ± standard errors (SEM), which was used to analyze the BBIX, qPCR and protein grayscale values. Statistical significance was calculated using Student’s *t*-test for individual paired comparisons or one-way ANOVA. For individual comparisons of multiple groups, Student–Newman–Keuls *post-hoc* test was used to calculate *p* value.

## 3 Results

### 3.1 IBDV field strain NN1172 infection result in thymic atrophy in SPF chickens

**Figure 2:**
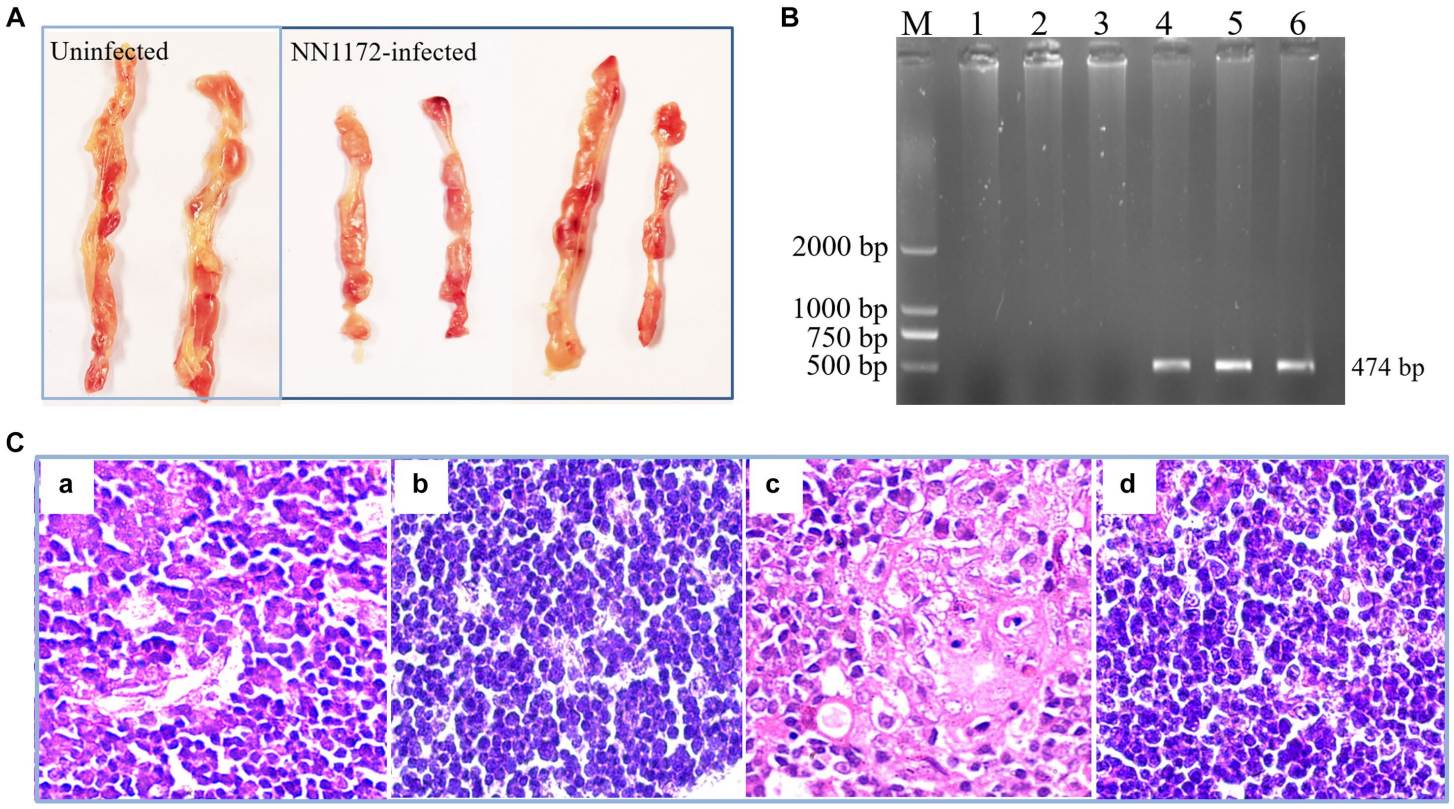
The Pathogenic characteristics of thymus in SPF chicken infected with NN1172. Thymus samples were randomly collected from the infected and uninfected chickens. **(A)** The morphology and gross lesion of the thymus. NN1172 infection induced severe thymus atrophy in the chickens at 3 dpi. **(B)** The results from RT-PCR detection based on IBDV-*v*VP2 in the thymus. M: 2,000 bp Marker, 1–3: uninfected group, 4–6: NN1172-infected chicken. And all the thymus samples from NN1172-infected chicken were positive for IBDV. **(C)** Thymic microscopical lesions in chicken infected with NN1172 strain at 3 dpi. **(a)** Infected thymic cortex (100×). **(b)** Uninfected thymic cortex (100×). **(c)** Infected thymic medulla (100×). **(d)** Uninfected thymic medulla (100×).

Firstly, we sought to confirm the pathogenicity of IBDV field strain NN1172 in the SPF chickens at 3 dpi. As showed in Table 2 and Figure 2, the BBIX evaluation showed that the BF and the thymus were all significantly atrophic in the NN1172-infected birds as compared to the uninfected group, as evidenced by both BBIX values being lower than 0.7 (Table 2) and the significantly atrophic appearance of the thymus in the NN1172-infected group (Figure 2A). RT-PCR detection further confirmed that the birds were infected with NN1172 (Figure 2B). We also conducted a comprehensive histological evaluation of thymic lesions, as depicted in Figure 2C. In the cortex, a noticeable alteration in lymphocyte morphology was observed in the infected group (Figure 2Ca), characterized by dark round or crescent-shaped homogeneous nuclei and sparsely stained, faint cytoplasm when compared to the uninfected group (Figure 2Cb). Moreover, the infected group exhibited prominent lymphocyte depletion, with a majority of cells being lysed (Figure 2Ca). Similarly, in the meulla, lymphocytes displayed altered morphology, and extensive cell lysis was evident in the infected group (Figure 2Cc) as compared to the uninfected control (Figure 2Cd). Notably, the extent and scope of the cell lysis were more extensive in the medulla than that in the cortex.

**Table 2:** The immune organ index and BBIX of the infected chicken.

	Bursa index (mg/g)	BBIX	Thymus index (mg/g)	BBIX
Uninfected group	3.511 ± 0.429	1	2.270 ± 0.042	1
Infected group	1.621 ± 0.067**	0.461	1.546 ± 0.050**	0.680

Note: Data are expressed as the mean index ± SD (*n* = 9 chicken and above per group). **p < 0.01 significant difference between NN1172-infected group and uninfected group.

### 3.2 Overview of RNA-seq and proteomic data

**Figure 3:**
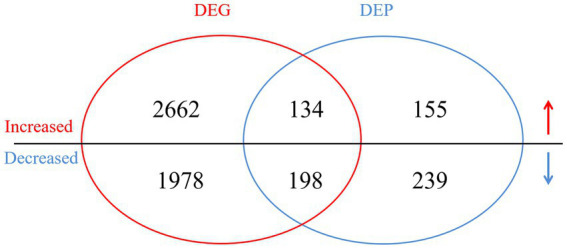
The Venn diagram of the differentially expressed genes (DEGs) and proteins (DEPs) in the infected vs. uninfected chickens. DEGs with a cut-off of 1.5-fold change and value of *p* < 0.05 between infected group and uninfected group were defined. DEPs with a cut-off of 1.2-fold change and *p*-value < 0.05 between infected group and uninfected group were defined. The overlap between the Venn diagram of the DEGs and DEPs revealed the differentially expressed genes in both mRNA and protein level.

In the transcriptomic analysis, 48,627,561 and 51,401,146 clean reads were detected in the NN1172-infected and uninfected groups, respectively (Supplementary Table S1). Based on a threshold of fold change (FC) ≥ 1.5 and *p*-value < 0.05, a total of 4,972 differentially expressed genes (DEGs) were identified between infected and uninfected groups, with 2,796 were up-regulated and 2,176 were down-regulated (Figure 3; Supplementary Table S2). In the proteomic analysis, a total of 394,009 spectra were identified, with 135,882 unique spectra were strictly matched to 67,227 peptides and further mapped to 20,890 unique proteins (Supplementary Table S3). Using a significance threshold of FC ≥ 1.2 and *p*-value < 0.05, 726 differentially expressed proteins (DEPs) were identified between infected and uninfected groups, comprising 289 up-regulated proteins and 437 down-regulated proteins (Figure 3; Supplementary Table S4). When the RNA-seq data were compared with the proteomic data, 359 genes were found to be differentially expressed at both the mRNA and protein levels. Among these genes, 134 were consistently up-regulated, 198 genes were consistently down-regulated, whereas the remaining 27 genes were inconsistently expressed at both of the mRNA and protein levels (Figure 3; Supplementary Table S5).

### 3.3 The gene ontology enrichment analysis suggests potential impacts on the structural integrity and functional dynamics of thymocyte infected by NN1172

**Figure 4:**
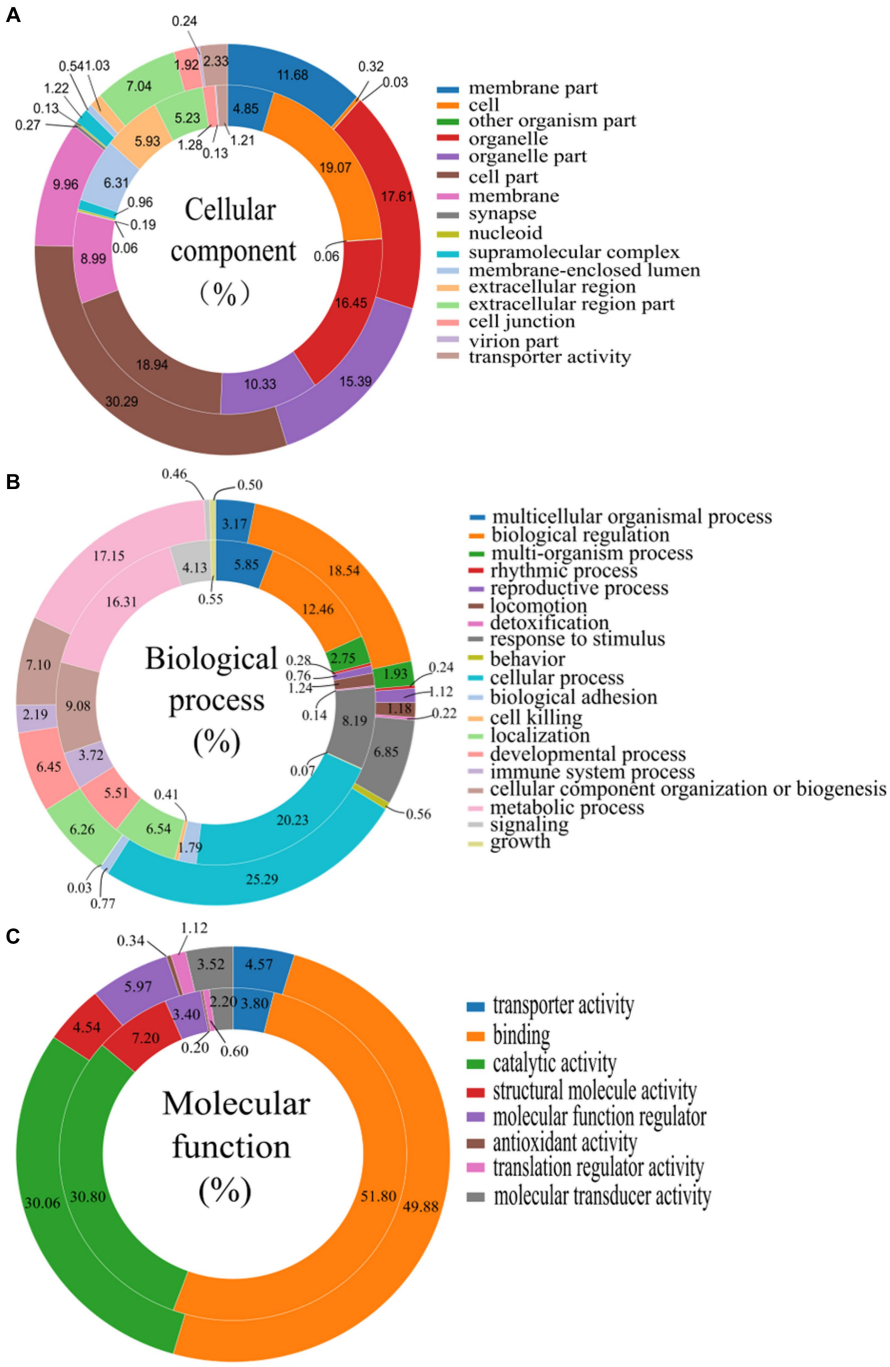
Gene ontology (GO) categories assigned to the differentially expressed genes (DEGs, outer cycle) and proteins (DEPs, inner cycle) in the thymus of chicken infected with NN1172 vs. uninfected control. **(A)** Percentage of cellular components. **(B)** Percentage of biological process. **(C)** Percentage of molecular function. The differentially expressed genes were classified into cellular component, molecular function, and biological process by WEGO (Web Gene Ontology Annotation Plot) according to the GO terms.

The GO analysis of all the DEGs and DEPs in chicken thymus of NN1172-infected versus uninfected was shown in Figure 4. Within the cellular components category, DEGs and DEPs were assigned to at least a spectrum of 16 categories. The majority of DEGs and DEPs were enriched in “cell part” (30.29% of genes and 18.94% of proteins), “organelle” (17.61% of genes and 16.45% of proteins), and “organelle part” (15.39% of genes and 10.33% of proteins). A smaller portion of DEGs and DEPs were located in “membrane part” (11.68% of genes and 4.85% of proteins), “membrane” (9.96% of genes and 8.99% of proteins), and “extracellular region part” (7.04% of genes and 5.23% of proteins). Only a limited number of DEGs and DEPs were observed in categories such as “transporter activity,” “cell junction,” “supramolecular complex,” “extracellular region,” “membrane-enclosed lumen,” “cell,” “synapse,” and “nucleoid.” In the context of biological processes, DEGs and DEPs were involved in 19 categories. The majority of these were significantly enriched in “cellular process” (25.29% of genes and 20.23% of proteins), “biological regulation” (18.54% of genes and 12.46% of proteins), and “metabolic process” (17.15% genes and 16.31% proteins). A smaller portion of the DEGs (ranging from 8.19 to 3.17%) and DEPs (ranging from 9.08 to 5.51%) were enriched in processes like “cellular component organization or biogenesis,” “responses to stimulus,” “developmental processes,” “localization,” and “multicellular organismal process.” A very limited portion of the DEGs (ranging from 4.13 to 0.03%) and DEPs (ranging from 3.72 to 0.07%) were associated with “immune system processes,” “multi-organism process,” “locomotion,” “reproductive process,” “biological adhesion,” “behavior,” “growth,” “signaling,” “rhythmic process,” “detoxification,” and “cell killing.” In terms of molecular function, DEGs and DEPs were categorized into 8 functional groups. The majority were significantly enriched in “binding” (49.88% of genes and 51.80% of proteins) and “catalytic activity” (30.06% of genes and 30.80% of proteins). The remaining DEGs and DEPs were linked to various molecular functions, including “transporter activity,” “structural molecule activity,” “molecular transducer activity,” “translation regulator activity,” and “antioxidant activity.”

### 3.4 KEGG pathway enrichment analysis of DEGs and DEPs revealed extensive immune activation and cell cycle/metabolism disorder in the thymus infected by NN1172

**Figure 5:**
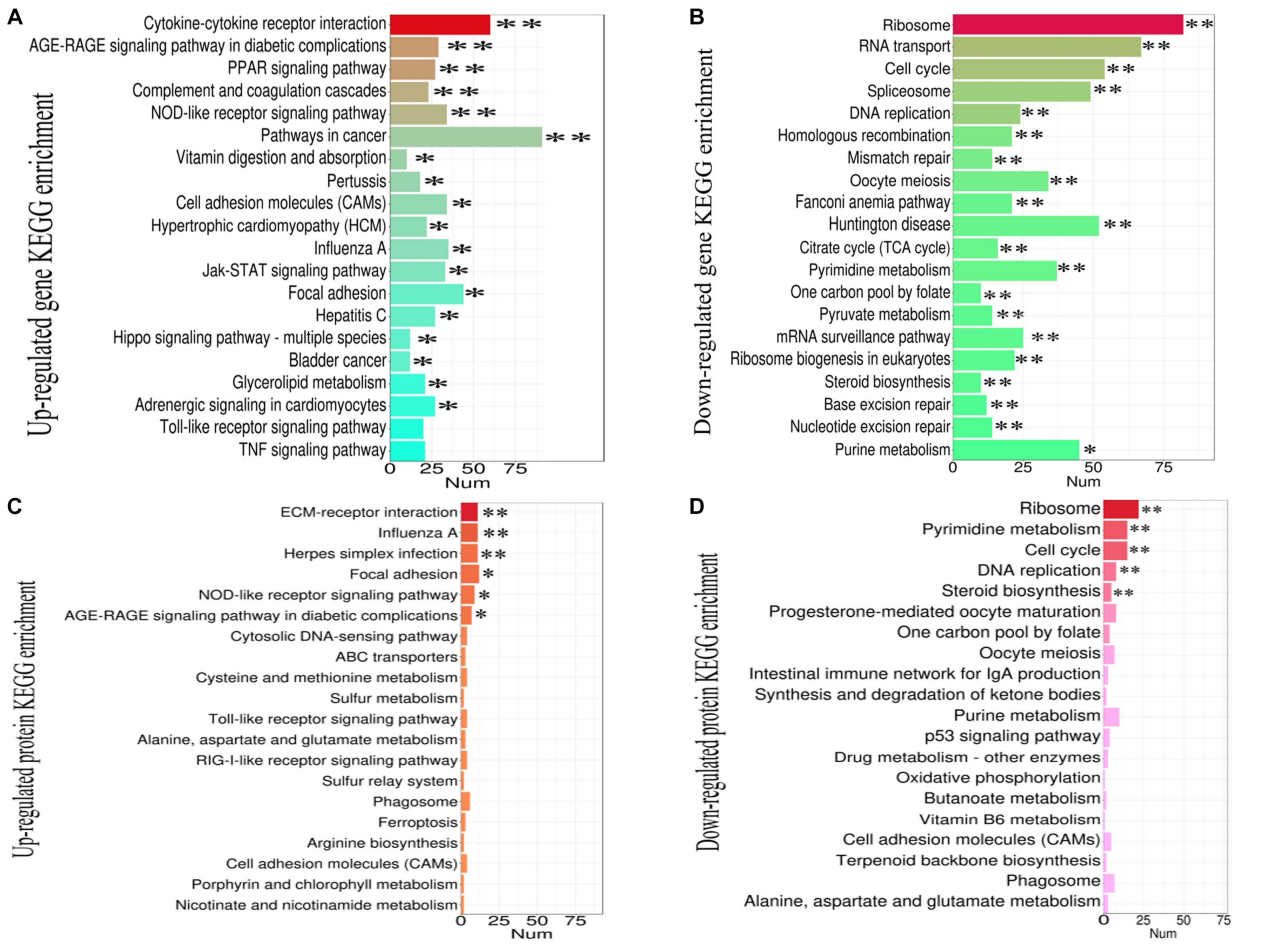
The KEGG pathway enrichment analysis of DEGs and DEPs in the thymus (chicken infected by NN1172 vs. uninfected control). **(A)** Up-regulated transcripts. **(B)** Down-regulated transcripts. **(C)** Up-regulated Proteome. **(D)** Down-regulated Proteome. The X-axis represents the number of genes or proteins. Y-axis represents the KEGG pathway. *p < 0.05 or **p < 0.01 represents significant difference in the KEGG pathway enrichment between chicken infected by NN1172 vs. uninfected control.

The DEGs and DEPs undergoing up-or down-regulated were subjected to KEGG enrichment analysis to further identify the contribution of the cellular factors that involved in the pathogenicity in the thymus that caused by NN1172. The top 20 enriched pathways were selected as showed in Figure 5. In Figure 5A, the up-regulated DEGs were significantly enriched in 18 pathways. Among them, 12 pathways, which included PPAR signaling pathway, AGE-RAGE signaling pathway in diabetic complications, Vitamin digestion and absorption, Cell adhesion molecules (CAM), Hypertrophic cardiomyopathy (HCM), Glycerolipid metabolism, Adrenergic signaling in cardiomyocytes, Jak–STAT signaling pathway, focal adhesion, hippo signaling pathway-multiple species, pathway in cancer, bladder cancer, were related to regulation of cell proliferation, metabolism and tissue damage. Whereas, six pathways, including cytokine-cytokine receptor interactions, complement and coagulation cascades, NOD-like receptor signaling pathway, Influenza A, Pertussis and Hepatitis C are directly associated with immune activation. In Figure 5B, the down-regulated DEGs were significantly enriched in all the top 20 pathways, the primarily involved in key cellular processes like protein synthesis (Ribosome, ribosome biogenesis in eukaryotes), DNA replication and repair (DNA replication, Homologous recombination, Mismatch repair, base excision repair, nucleotide excision repair, Fanconi anemia pathway), mRNA synthesis (Spliceosome, mRNA surveillance pathway mRNA, RNA transport, Huntington disease), Energy metabolism (TCA cycle, steroid biosynthesis), nucleotide metabolism (TCA cycle, Pyrimidine metabolism, Pyruvate metabolism, purine metabolism, one carbon pool by folate) and cell proliferation (Oocyte meiosis, Cell cycle) activity. Figure 5C revealed that the up-regulated DEPs were significantly enriched in 6 pathways, with 4 pathways-the influenza A, NOD-like receptor signaling pathway, focal adhesion, and AGE-RAGE signaling pathway in diabetic complications were consistent with that of DEGs, the other two pathways were ECM-receptor interactions and herpes simplex infection, all of which were related to immune activation and tissue damage. In Figure 5D, the down-regulated DEPs were significantly enriched in 5 pathways, encompassing ribosomes, pyrimidine metabolism, cell cycle, DNA replication and steroid biosynthesis, and all consistent with the DEGs, collectively highlighting the disruptions in protein synthesis, DNA replication and repair, nucleotide metabolism, Energy metabolism and cell proliferation.

### 3.5 Analysis of NN1172-modified genes/proteins

**Figure 6:**
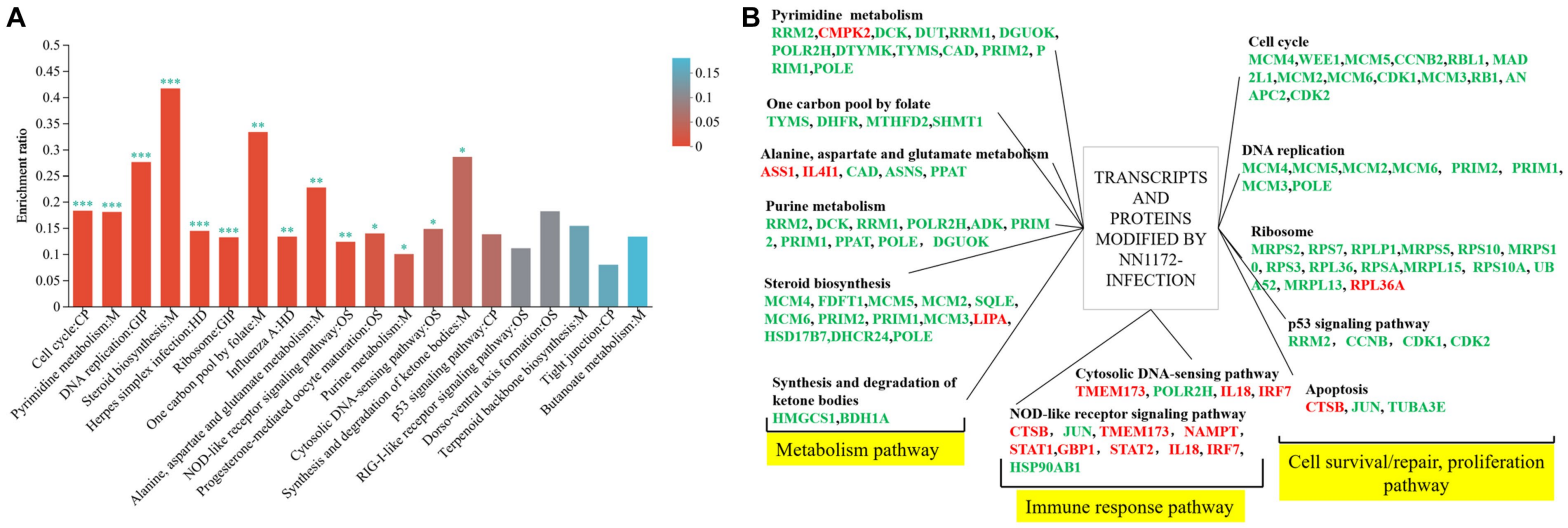
Functional classification of transcripts and proteins in the thymus of chicken infected by NN1172. **(A)** 359 DEGs/DEPs KEGG enrichment. The x-axis indicates the pathway; y-axis indicates the enrichment rate [the number of proteins enriched in the pathway (Protein number)/the number of proteins annotated to pathway (Background number)]. The higher enrichment ratio, the greater degree of enrichment. The color gradient of the column corresponds to different p-adjust ranges. *p < 0.05, **p < 0.01, and ***p < 0.001 represent significant difference in the KEGG pathway enrichment between chicken infected by NN1172 vs. uninfected control. **(B)** The co-expressed genes in both mRNA and protein level induced by NN1172 infection in the thymus were manual classified into functional categories. Upregulated genes were shown in red and downregulated genes in green. The differentially expressed genes with no significant difference in the corresponding pathway were not listed in the figure.

In order to further explore the functional genes/proteins that modified by NN1172 in the thymus, the 359 genes differentially expressed at both the mRNA and protein levels were further analyzed by the KEGG pathway (Figure 6A; Supplementary Table S6). Totally 14 pathways which were mainly contributed to cell proliferation and death (including cell cycle\DNA repair\translation), metabolism, and innate antiviral immunity were significantly enriched. Further, a manual categorization of these 359 genes based on their presumed biological function unveiled a multifaceted transcriptional and translational response triggered by NN1172 infection in the thymus (Figure 6B). Among the functions that were most affected were metabolism pathway, cell survival/repair, replication and death pathway, which included the most down-regulated genes. Additionally, NN1172 infection caused gene expression changes related to antiviral immune response which included the most up-regulated genes.

### 3.6 Network inference analysis revealed the potential key mediators of NN1172-induced pathogenicity in the thymus

**Figure 7:**
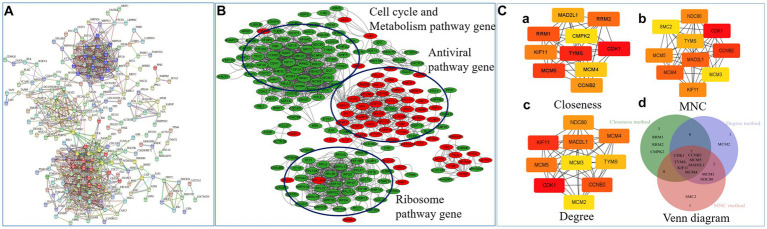
Network inference analysis of NN1172-modified genes/proteins. **(A)** The PPI network of 359 genes/proteins were constructed by online STRING database. A red line indicates gene fusions interaction, a blue line indicates know interaction from curated database and pink line indicates interaction that was experimentally determined. Others include textmining (yellow line), co-expression (black line). **(B)** The PPI network visualized by Cytoscape. Red represents up-regulated genes, Green represents down-regulated genes. **(C)** Top 10 hub gene network. **(a)** The top 10 genes derived from the Closeness method were chosen using the CytoHubba plugin. **(b)** The top 10 genes derived from the Maximum neighborhood component method were chosen using the CytoHubba plugin. **(c)** The top 10 genes derived from the Degree method were chosen using the CytoHubba plugin. **(d)** Venn diagram showing overlap hub genes (totally Seven Hub genes) between the different algorithms in the CytoHubba plugin.

In order to unravel potential key mediators of NN1172-induced pathogenicity within the thymus, a comprehensive analysis of the interactive relationships among the 359 differentially expressed genes/proteins were conducted. By using String software,^13^ and a detailed Protein–protein interaction (PPI) network (Figure 7A) was constructed and visually represented it using Cytoscape (Figure 7B). The results showed that the PPI network consisted of 328 nodes and 862 edges, which was further distinctly segregated into three clusters: cell cycle and metabolic pathways, ribosomal pathways, and antiviral pathways. To explore the crucial mediators, we utilized various calculation methods in the CytoHubba plugin within Cytoscape. The top 10 genes identified by each method (Closeness method in Figure 7Ca, MNC method in Figure 7Cb, Degree method in Figure 7Cc) revealed a consistent set of seven core hub genes (Figure 7Cd). These genes exhibited significant connectivity with others within the PPI network and played a central role in the pathogenicity induced by NN1172 in the thymus. Table 3 provides detailed annotations for these seven core hub genes, all of which were found to be down-regulated. Notably, CDK1 (cyclin-dependent kinase 1) displayed the most substantial down-regulation, with a score of 84.20357, followed by TYMS (thymidylate synthase) with a score of 82.00476, and MCM5 (DNA replication licensing factor 5) with a score of 80.0369. KIF11 (kinesin-like protein), CCNB2 (G2/mitotic-specific cyclin-B2), MAD2L1 (mitotic spindle assembly checkpoint protein MAD2A), and MCM4 (DNA replication licensing factor) presented lower scores, hovering around 77.

**Table 3:** The annotation of the core Hub genes identified by using different algorithms in the Cytohubba plugin.

Rank	Gene	Annotation	Score	Regulation
1	CDK1	Cyclin-dependent kinase 1 isoform X1	84.20357	Down
2	TYMS	Thymidylate synthase	82.00476	Down
3	MCM5	DNA replication licensing factor 5	80.0369	Down
4	KIF11	Kinesin-like protein KIF 11	77.7869	Down
5	CCNB2	G2/mitotic-specific cyclin-B2 isoform X1	77.70357	Down
6	MAD2L1	Mitotic spindle assembly checkpoint protein MAD2A	77.12024	Down
7	MCM4	DNA replication licensing factor 4	77.0369	Down

Sorted by score size from closeness method.

### 3.7 Validation of DEGs by RT-qPCR and DEPs by western-blot analysis

**Figure 8:**
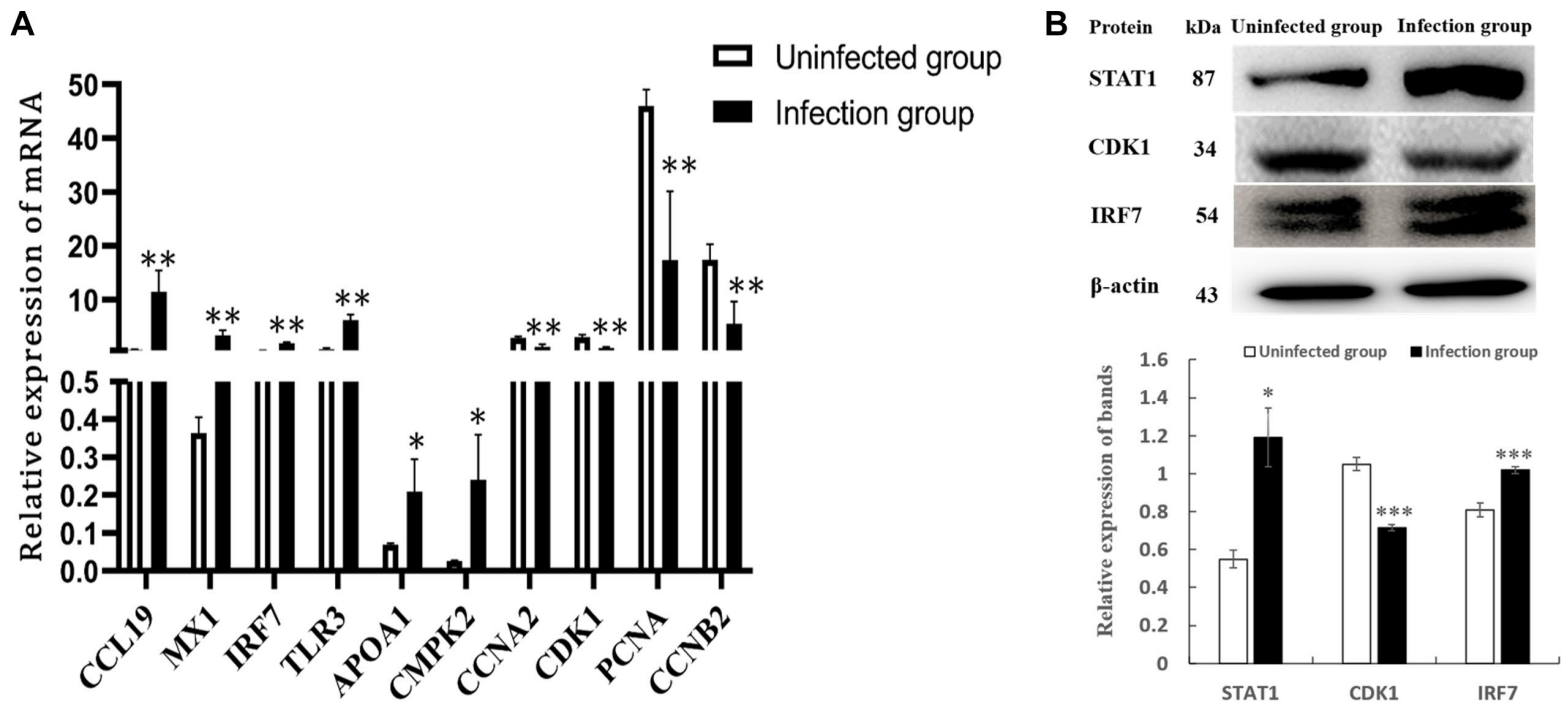
Validation of differentially expressed genes. **(A)** DEGs by RT-qPCR. **(B)** DEPs by western-blot analysis. *p < 0.05 significant difference between infection group and uninfected group. **p < 0.01 significant difference between infection group and uninfected group.

After conducting a rigorous conditional screening process to identify differential genes, 10 genes were randomly selected for RT-qPCR validation. The results showed that gene expression was significantly changed. Specifically, the expression levels of CCL19, IRF7, MX1, TLR3, APOA1, and CMPK2 were up-regulated, whereas CCNA2, CDK1, PCNA, and CCNB2 were down-regulated when compared to the uninfected group (Figure 8A). Importantly, these changes in gene expression within IBDV infection group were consistent with the patterns observed in the transcriptomic data. Further, in the protein level, the expression of STAT1 and IRF7 were obviously up-regulated, while CDK1 was down-regulated in the NN1172-infected group (Figure 8B), which were also consistent with that from proteomic data.

## 4 Discussion

As a bi-segmented dsRNA virus, IBDV undergoes extensive gene mutations, segment reassortment and homology recombination in the genome and result in the changes in the virulence and antigenicity of the epidemic strains in the field (4, 11, 35, 43–46). To date, IBD is still one of the most economically important immunosuppressive disease affecting young chickens aged 3–6 weeks old. Previous investigations into the virus’s pathogenesis have predominantly focused on host-pathogen interaction using Omics studies, both *in vitro* and *in vivo*, primarily involving host cell type such as fibroblast, DF-1 cells and bursal cells (16, 19–21, 47, 48). However, the thymus, serving as the central immune organ responsible for T cell maturation and differentiation, has been confirmed as an important target tissue in IBDV pathogenesis (6, 11). To date, our understanding of the molecular mechanisms underlying IBDV-induced pathogenicity in the thymus remains limited. In this study, we have, for the first time, elucidated the potential regulatory mechanisms underlying the pathogenicity of a vvIBDV field strain in the thymus of SPF chicken, employing an integrated approach that combines transcriptomic and proteomic analyses.

Programmed cell death is one of the pivotal host defense mechanism to minimize viral spread, and its role in IBD pathogenesis has been extensively recognized (12, 49). Following viral infection, the activation of programmed cell death pathways leads to two distinctive outcomes: suicidal cell death, or apoptosis, and lytic cell death. Simultaneously, uninfected neighboring cells are alerted to initiate antiviral responses (50). Notably, several DNA and RNA viruses target critical cell cycle regulators to manipulate cellular conditions favoring viral replication. Some viruses even have the ability to arrest cells in a specific phase of the cell cycle conducive to their replication (51), resulting in cellular demise by the activation of apoptotic or non-apoptotic cell death programs. Consistently, the thymus infected by NN1172 exhibited atrophy as early as 3dpi as demonstrated by the BBIX value falling below 0.7. Histological examinations further showed severe thymocytes lysis and depletion. Our integrated transcriptomic and proteomic analyses consistently identified a significant down-regulation in genes/proteins associated with cell cycle, DNA replication and ribosome pathway. Notably, when the common proteins were further analyzed, the down-regulated genes/proteins were mainly functionalized to metabolism pathway and cell survival/repair, proliferation pathway (Figure 6B) or gathered in cell cycle and metabolism pathway cluster and ribosome pathway cluster by PPI method (Figure 7). Further hub gene analysis identified seven key genes involving in this disruption, including CDK1, TYMS, MCM5, KIF11, CCNB2, MAD2L1, and MCM4, all of which are intricately linked to cell survival and replication pathway. Taken together, these findings highlight the substantial impact of IBDV infection on cell survival mechanisms, coupled with significant disruptions in metabolic processes. These alterations have the potential to fundamentally modify the underlying biological mechanisms within the thymus, which in turn would affect the T cell immune response, favoring for IBDV replication.

The induction of apoptosis depends on the cellular context: conflicting signals for cell proliferation and cell cycle arrest may result in cell death (52). Usually, at the core of the cell cycle, CDKs drive cells through the different phases of the cell cycle. NN1172-infection leads to the down-regulation of many genes related to cell cycle and DNA replication genes, including MCM 2 ~ 6, CDK1 ~ 2, CCNB2, ANAPC2, MAD2L1, WEE1, RBL1, RB1. We noticed that these genes were included in all the stage of cell cycle. Generally, key regulatory proteins that control cell cycle progression are cyclins and CDKs (51). CDKs represent a family of serine/threonine protein kinases activated at specific cell cycle points. In mammalian cells, five CDKs that have been associated with cell cycle progression: CDKs 4 and 6, active in early G1 phase; CDK2, active in late G1 and S phase; and CDK1, active in G2 and M phases. Notably, CDK1 has the capacity to substitute for other CDKs and is sufficient for driving the mammalian cell cycle (53). The activity of CDKs is tightly regulated and requires the expression of activating cyclins and phosphorylation of the cyclin-CDK complex. Interestingly, in the NN1172-infected thymus, CDK1 and its dependent cyclin, CCNB2 emerged as hub genes. CCNB2 is known to be expressed during mitosis, CCNB2-CDK1 can regulate the remainder of mitosis. Several viruses, including serotype 3 Reovirus, Murine gamma herpesvirus 68 virus, HSV-1, HPV1, exert control over the G2/M checkpoint and progression through G2 and M phases by influencing DNA replication, CDK1 activity, or the cyclin B1-CDK1 complex (54–57). Given the down-regulation of hub gene CDK1, CCNB2, and MCM4 in the NN1172-infected thymus, along with the down-regulation of pathways related to mismatch repair, DNA replication, base excision repair, and nucleotide excision repair, we speculate that NN1172 infection may induce incomplete genome replication and insufficient cyclin B1-CDK1 complex activity, leading to G2 arrest and prevents entry into mitosis. CDK1 has also been linked to the control of protein synthesis during M phase (58, 59). Besides the down regulation of CDK1, our findings indicate a significant down-regulation of genes associated with the protein synthesis system as evidenced by extensive down-regulated genes enriched in the ribosome-related pathway, RNA transport, spliceosome, ribosome biosynthesis in eukaryotes. Recently, Wu and Hur (60) confirmed that the CDK1-cyclin B1 complex phosphorylates VP1, thereby facilitating IBDV replication. Collectively, these results suggest that NN1172 may arrest cell cycle at G2/M stage, which may enhance virus replication, thymus damage and disease progresses.

Following IBDV infection, previous research, as indicated by Ou et al. (15) in their transcriptome analysis, has consistently reported a robust immune response in the bursa during the early stages of infection. Consistently, we also found a similar response in the thymus at 3dpi as evidenced by the pronounced up-regulation of genes enriched in pathways such as cytokine-cytokine receptor interactions, complement and coagulation cascades, NOD-like receptor signaling pathway, Influenza A, Pertussis, and Hepatitis C. Interestingly, when the up-regulated DEPs were analyzed, only influenza A and NOD-like receptor signaling pathway were significantly enriched accordingly (Figures 5A,C). Further, the up-regulated proteins that involved in these two pathways were mainly included interferon-induced proteins, such as interferon-induced helicase C domain-containing protein 1 (IFIH1, also known as a melanoma differentiation-associated protein 5 - MDA5), interferon-induced GTP-binding protein Mx, interferon-induced and double-stranded RNA-activated protein kinase EIF2AK2 (also named protein kinase R, PKR), interferon regulatory factor 7 (IRF7), Interleukin-18 (IL-18), signal transducer and activator of transcription 1 and 2 (STAT1, STAT2) (Supplementary Table S6). These proteins are all key proteins that mediate the host’s innate antiviral immune response upon viral infection (61–64). As members of the RLR family, MDA5 serve as dsRNA sensors with different specificity to recognize infection by many RNA viruses (65, 66). Upon viral dsRNA binding, MDA5 activates the common downstream adaptor molecule MAVS. Activated MAVS then recruits multiple signaling molecules, including TRAFs, TBK1, and IRF3/7, leading to the transcriptional upregulation of type I interferons and other proinflammatory cytokines (67, 68). Studies have confirmed that chicken MDA5 is activated by IBDV infection, leading to the up-regulation of IRF-3, IFN-β, IL-18, PKR, OAS, and Mx in a virus-dose-dependent manner (29, 48, 69, 70). Taken together with our results, the innate antiviral response induced by MDA5 also plays important role on the pathogenicity of vvIBDV in the thymus in the early-stage infection.

Interestingly, when the proteins were further analyzed, we found that besides the up-regulation of IL-18, we could not detect the up-regulation of IL-12. In Nakanishi et al.’s review (71), IL-18 normally can enhance the IL-12-driven Th1 immune responses after stimulation, however, it can also stimulate Th2 immune responses in the absence of IL-12; further, overproduction of IL-18 is responsible for tissue injury. Considering the histological changes observed in the thymus, it is conceivable that a predominant Th2 cell response may be underway during this stage of infection, potentially exacerbating disease progression, which consistent with our previous research, where we reported that Th2 cell response plays important role in the IBD progression (72). In this study, we also detected cytokines, chemokines at the mRNA level as evidenced by up-regulation genes enriched in the cytokine-cytokine receptor interaction pathway which is consistent with many researches (15, 16). This suggests that IBDV infection stimulate a strong innate immune response at the mRNA level, even within the thymus. However, it’s noteworthy that most of these genes could not be detected on protein level in the thymus at 3dpi, indicating potential limitations in the transcriptome-level analysis.

It is noteworthy that the IBDV field strain, NN1172, employed in this study, is a naturally reassortant strain and has been confirmed to be a vv field strain that induces the most severe pathogenicity in both of the bursal and thymus tissues of commercial Three-Yellow chickens when compared to isolates with different kind of genome reassortant (11). In that study, NN1172 was found to increase the bursa/body weight (B/BW) and thymus/body weight (T/BW) ratiosin the commercial Three-Yellow chickens at 3dpi, followed by a subsequent decrease at 7 dpi. However, the disease induced by NN1172 progresses more rapidly in the SPF chickens than that in Three-Yellow chicken. In this study, NN1172 induced sever atrophy in the thymus and bursa, accompanied by profound lymphoid cell lysis and depletion in both the cortex and medulla region of the thymus in SPF chicken as early as 3 dpi. Inoue’s (12) research found that chicken thymus infection with IBDV leads to atrophy, which coincides with our research results. Notably, chicken’s genetic background has been confirmed to influence the susceptibility of IBDV with leghorn-type SPF chickens showed the highest susceptibility to IBDV among any other genetic background chickens (6). Additionally, it is well-documented that vvIBDV strains exacerbate susceptibility in leghorn-type SPF chickens; thymus atrophy, in particular, has been associated with the acute phase of the disease and might be indicative of the virulence of the isolate (13). These collective findings provide further compelling evidence that NN1172 is a vvIBDV strain.

## 5 Conclusion

This study reveals, for the first time, the potential molecular mechanism underlying severe thymocyte lysis and depletion induced by vvIBDV field strain in infected SPF chickens. The infection with vvIBDV NN1172 leads to extensive differentially expressed genes at both the mRNA and protein levels within the thymus, encompassing various categories related to cellular components, biological processes, and molecular functions. The host’s response is predominantly characterized by severe disruptions in pathways associated with cell survival, repair, proliferation, and metabolism, which may contribute to thymocyte lysis and depletion. Furthermore, seven genes (*CDK1, TYMS, MCM5, KIF11, CCNB2, MAD2L1*, *and MCM4*) have been identified as core hub genes involved in cell-cycle regulation, potentially serving as mediators in the pathogenesis triggered by NN1172 infection within the thymus. The findings of the new pathways and genes in the IBDV-infected thymus will provide insight into the pathogenesis of IBDV in chickens.

## Data availability statement

The RNA seq and Protein data presented in the study are publicly available. The RNA seq data can be found here: NCBI repository, accession number PRJNA1054295. The protein data can be found here: iProx repository, accession number IPX0007777000.

## Ethics statement

The animal study was approved by the Animal Experimental Ethical Committee of Guangxi Minzu University and the approval code is 202010608001. The study was conducted in accordance with the local legislation and institutional requirements.

## Author contributions

JC: Methodology, Writing – original draft. WW: Formal analysis, Methodology, Writing – original draft. SL: Methodology, Writing – original draft. ZW: Data curation, Writing – original draft. WZ: Data curation, Writing – original draft. TN: Data curation, Writing – original draft. YL: Formal analysis, Writing – review & editing. HL: Formal analysis, Writing – review & editing. PW: Writing – review & editing. XH: Conceptualization, Funding acquisition, Writing – review & editing.

## Footnotes

^1^ https://github.com/OpenGene/fastp

^2^ http://tophat.cbcb.umd.edu/

^3^ http://ccb.jhu.edu/software/stringtie/

^4^ ftp://ftp.ncbi.nlm.nih.gov/blast/db/

^5^ http://web.expasy.org/docs/swiss-prot_guideline.html

^6^ http://pfam.xfam.org/

^7^ http://www.ncbi.nlm.nih.gov/COG/

^8^ http://www.geneontology.org/

^9^ http://www.genome.jp/kegg/

^10^ http://deweylab.biostat.wisc.edu/rsem/

^11^ http://bioconductor.org/packages/stats/bioc/DESeq2/

^12^ https://www.blast2go.com/program

^13^ https://cn.string-db.org/
